# Understanding and Overcoming the Sticking Point in Resistance Exercise

**DOI:** 10.1007/s40279-015-0460-2

**Published:** 2016-01-12

**Authors:** Justin Kompf, Ognjen Arandjelović

**Affiliations:** Kinesiology Department, Park Center, State University of New York at Cortland, Cortland, NY 13045 USA; School of Computer Science, St Andrews University, St Andrews, Fife KY16 9SX Scotland, UK

## Abstract

In the context of resistance training the so-called “sticking point” is commonly understood as the position in a lift in which a disproportionately large increase in the difficulty to continue the lift is experienced. If the lift is taken to the point of momentary muscular failure, the sticking point is usually where the failure occurs. Hence the sticking point is associated with an increased chance of exercise form deterioration or breakdown. Understanding the mechanisms that lead to the occurrence of sticking points as well as different training strategies that can be used to overcome them is important to strength practitioners (trainees and coaches alike) and instrumental for the avoidance of injury and continued progress. In this article we survey and consolidate the body of existing research on the topic: we discuss different definitions of the sticking point adopted in the literature and propose a more precise definition, describe different muscular and biomechanical aspects that give rise to sticking points, and review the effectiveness of different training modalities used to address them.

## Key Points

**Table:** 

Existing definitions of the sticking point (or region) in the literature fail to capture the phenomenon of practical interest adequately.
Thorough analysis of the factors underlying the development of sticking points shows the aetiology to be highly multifactorial, demanding careful case-by-case exercise prescription.

## Introduction

The “sticking point” (or sometimes the “sticking region”) is a concept commonly used in the context of resistance training [1–3]. Broadly speaking it refers to the part of the range of motion (ROM) in a resistance exercise in which a disproportionately large increase in the difficulty to continue the lift is experienced. If the exercise is performed to exhaustion, failure is often experienced in the vicinity of the sticking point. Hence, two important practical concerns can be immediately observed. The first of these regards performance. If the sticking point is the proverbial weakest link in the execution of an exercise, it is the limiting factor, which can have a profound effect on the load an athlete can employ in training or, in the case of athletes who compete in sports that inherently involve weight lifting (e.g. weight- and powerlifting), can directly impact competitive achievement. The second important concern is that of safety and injury prevention. A disproportionate increase in the difficulty of the lift, often coupled with a biomechanically weak ROM in which the sticking point occurs [4], increases the chance of exercise form breakdown and consequently injury. Therefore, understanding the multitude of factors that play a role in the development of sticking points [5, 6] as well as different strategies that a trainee can employ to remedy the associated weaknesses is of major importance to strength training practitioners.

In the present article we review different physiological and biomechanical aspects of resistance exercise which are of interest in this context, place these into practical context using examples from observational studies from the literature, and survey the body of evidence behind different relevant training methodologies. Relevant literature was collected by searching Google Scholar^1^ and PubMed^2^ databases, initially using queries comprising combinations of search terms ‘sticking point’, ‘sticking region’, ‘resistance’, ‘strength’, ‘powerlifting’, ‘weightlifting’, ‘training’, and ‘bodybuilding’, as well as by directly accessing works referenced by any of the already collected publications. Since ballistic exercises by their very nature include periods during which the athlete exerts little or no force against the load, the analysis of sticking points (and even the very definition thereof) in this context requires a somewhat different treatment from that in the context of conventional exercises; hence in this work we restrict our consideration to non-ballistic exercises.

## The Sticking Point

Although the concepts of the sticking point and sticking region are pervasive in sports and exercise science research, what is precisely meant by these terms is seldom discussed in detail in the published academic literature. Rather, in most instances, loose and semi-colloquial definitions are given. A review of different definitions encountered in the literature reveals several important problems with this approach. First, the seemingly subtle differences in the range of definitions that can be found have a profound effect on the analysis of the phenomena of interest, including their aetiology or the means of overcoming the associated performance bottlenecks; indeed, this is one of the likely reasons for the apparently contradictory findings reported in empirical studies. Second, when analysed with some scrutiny some of the popular definitions are readily found not to correspond well to the understanding of these concepts as they are used in strength training practice, often failing to capture important phenomena of interest while including in their scope phenomena that are of little relevance to either optimal athletic performance or the safety of the athlete. To address this weakness of the existing literature, in this section we discuss how the sticking point should be defined to inform the analysis in the most useful manner. We begin by reviewing some of the most often used definitions in the literature, highlight their strengths and weaknesses, and emerge with a precise definition that places the issue on a firm and rigorous scientific footing.

In the discussion of the sticking point, many of the authors focus their attention on the velocity of the load that is being lifted (e.g. barbell, dumbbell, weight stack). One of the most widely cited definitions of the sticking point is that voiced amongst others by Hales et al. [7] and McGuigan and Wilson [8] according to whom the sticking point is understood to be the point in the range of motion during an exercise at which the upward velocity of the load decreases or reaches zero. Notwithstanding its intuitive appeal, when examined with rigour this definition can be seen to be inadequate in several ways. Most obviously, an immediate corollary of the aforementioned definition is that any lift, no matter how effortlessly completed, has to have a sticking point—given that both at the beginning and the end of each lift the load is at rest, and its velocity cannot keep increasing or fail to decrease at some point. The definition can also readily be seen to lack sufficient precision for it does not state whether the sticking point is one where the velocity starts decreasing or where it reaches its minimum. The former does not appear to be a meaningful candidate since a mere reduction in velocity from its maximum does not ipso facto reflect a performance bottleneck as the load may still have substantial velocity. The point at which the velocity reaches its minimum, which was indeed proposed by Król et al. [9] and Madsen and McLaughlin [10] amongst others, also does not correspond to a performance limiting point in a lift since, by definition, the velocity thereafter increases, which means that at the point of minimum velocity the athlete is capable of supplying force sufficient to overcome the imposed resistance.

**Fig. 1:**
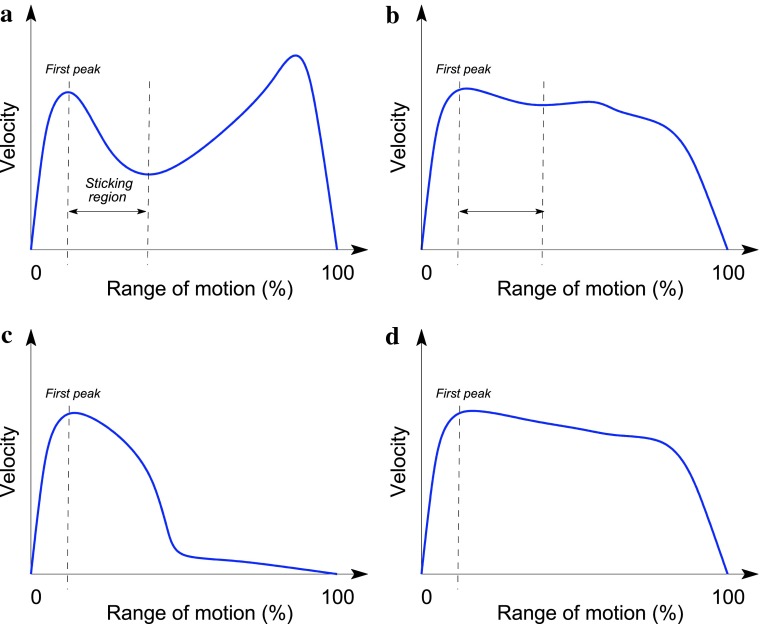
Defining the sticking point/region. **a** A conceptual illustration of a popular definition of the sticking region adopted by van den Tillaar et al. [11] amongst others. **b** Region identified as the sticking region despite the minimal drop in the velocity of the load. **c** The aforementioned definition identifies no sticking region despite the clear and rapid performance drop approximately half way through the lift. **d** An easily completed lift

Some of these concerns are addressed by various authors by their rejection of the notion of a single sticking point in favour of a somewhat more flexible concept of a sticking region. Recent notable studies that fall under this umbrella include a number of publications by van den Tillaar et al. [11, 12] and Escamilla et al. [13] who define the sticking region as the part of the range of motion in an exercise between the first peak in the velocity of the load and its first local minimum thereafter, as illustrated in Fig. 1a. Although this approach avoids the difficulties associated with identifying a singular problematic point in a lift, focussing rather on a range of motion in a lift during which an athlete could be argued to struggle, the change in perspective does little with regard to the problem identified earlier and that stems from the lack of any quantitative criterion in deeming a range of motion a sticking region. As noted previously a mere reduction in the speed of the load from its very peak can hardly be sufficient; see the conceptual illustration in Fig. 1b. What is more, as evidenced by empirical data [3, 4] and predicted by computational models [14], since the region in which a slowdown of the load occurs (in general) varies throughout a set of repetitions (i.e. as fatigue accumulates), the definition of van den Tillaar et al. would lead to an inevitable conclusion that there is a whole series of sticking regions for a particular lifter in a given exercise, jointly possibly extending to include the entire range of motion. In addition to not facilitating the localisation of the actual performance-limiting factor in an exercise, this corollary suggests that the definition is at the very least not a particularly useful one.

Another important aspect in which all of the aforementioned definitions, i.e. both the sticking point definition of McGuigan and Wilson and its varieties as well as the sticking region definition of van den Tillaar et al., fail to capture the phenomenon of interest adequately concerns their inability to account for the possibility of a sticking point (or region) that occurs at the end of a lift, as illustrated conceptually in Fig. 1c. As in the example shown no local velocity minimum exists, as discussed explicitly by van den Tillaar et al. [11], no sticking region would be identified in this case. In other words, if their definition is accepted, both the aforementioned example in Fig. 1c and that in Fig. 1d result in the same conclusion that the athlete’s performance exhibits no sticking region. While this can be readily agreed upon in the latter case, it can be hardly accepted in the former. Note that merely including the end point of the lift as a special case of a minimum (seeing that the lift always ends with the load stationary) does nothing to resolve the problem, for although this would entail a sticking region corresponding to the second half of the lift in Fig. 1c, it would necessarily lead to the same outcome for the performance in Fig. 1d. As remarked earlier, the given definition of the sticking region again makes it impossible to distinguish between the two performances as it fails to include any quantitative criteria.

Rather than on the velocity of the load, the second group of definitions focusses on force (which is of course related to the rate of change in velocity), that is, the difference between the effective force exerted against the load by the lifter and the force that resists the movement (usually the weight of the load). Elliott et al. define the sticking point as one where the lifter experiences apparent difficulty in exerting effective force against the load [4]; a similar definition is adopted by García-López et al. [15] and Kulig et al. [16] amongst others. Much like before, although superficially appealing, this definition can be readily rejected as it leads to conclusions that do not reflect the nature of the phenomenon we wish to describe well. For example, given that muscular force reduces with the velocity of muscular shortening, the lifter may not be able to apply much force against the load because the load has a very high velocity; this leads to the bizarre conclusion that the sticking point is in the range of motion in which the bar moves most swiftly, i.e. with the greatest ease. The definition offered by the National Strength and Conditioning Association defines the sticking point as the weakest point in the range of motion of an exercise and clarifies that it probably occurs where the external resistance has the greatest mechanical advantage [17]. This definition is not readily reconciled with empirical observations such as that the sticking point at the end of the range of motion is commonly observed both in the bench press and the deadlift, say, yet in both of these cases these positions are biomechanically advantageous to the lifter [18, 19]. The focus on purely instantaneous biomechanics fails to capture the context of the lift including the accumulated fatigue as well as the force-velocity dependence (which we review in the next section), the importance of which was highlighted in previous work [16, 20].

### An Unambiguous Definition

To summarise our discussion above, many of the popular definitions of the sticking point and the sticking region suffer from some of the following key limitations or inconsistencies:

1. Failure to account for the possibility of the sticking point or region occurring at the beginning of a lift,
2. Failure to account for the possibility of the sticking point or region occurring at the end of a lift,
3. Identifying the sticking point in the range of motion where the bar has substantial velocity and is moving with relative ease,
4. Identifying the sticking point in the range of motion where the lifter can exert force substantially greater than the resistance,
5. Reliance purely on qualitative criteria and failure to account for any quantitative considerations,
6. Failure to account for the exercise context such as fatigue accumulation and the velocity of the load, and
7. Leading to a series of sticking points or regions across a set of repetitions, thereby resulting in a poorly localised performance bottleneck and little insight into how it may be corrected.

Motivated by these observations, in this work we argue that it is best to adopt the notion of a sticking point rather than a sticking region and propose to define it as the point at which failure occurs when exercise is taken to the point of momentary muscular failure. Different forms of this definition were previously adopted by various authors such as Blackburn and Morrissey [21] and Cotterman et al. [22]. To see how this definition overcomes the difficulties enumerated above, first observe that by its very nature it identifies a single, well-defined point in a lift (thereby addressing issue 7 in the list), which can be anywhere in the range of motion (thereby addressing issues 1 and 2). Considering that failure occurs at this sticking point, issues 3 and 4 are immediately addressed too, as are issues 5 and 6. In addition to being clearly and uniquely defined, and not leading to any of the listed problems, the proposed definition is also ipso facto the performance bottleneck.

## Understanding the Sticking Point

**Fig. 2:**
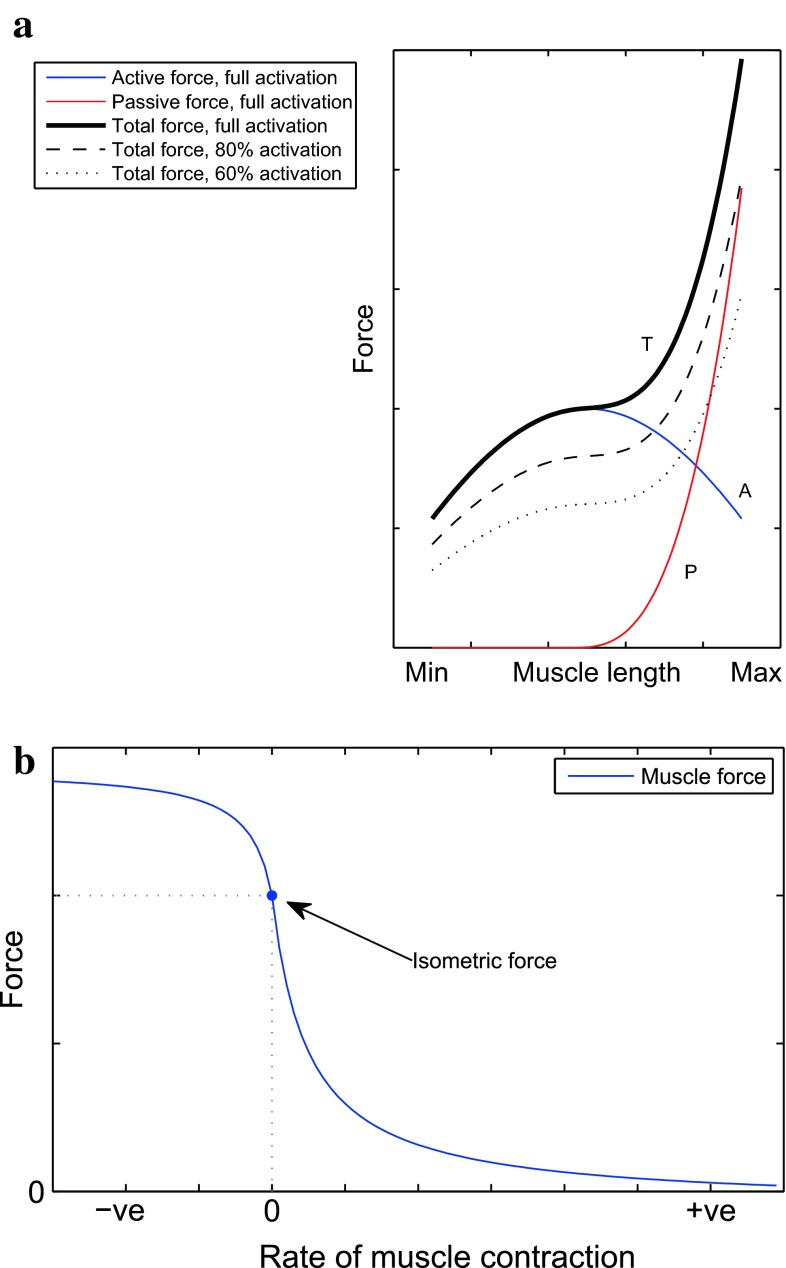
Muscle force modulation. **a** A typical force-length diagram (not to scale) for an isolated striated muscle [35]. Two components contributing to total force production (*T*, *black*) are shown: active (*A*, *blue*) and passive (*P*, *red*). Total forces for different levels of muscle activation are shown in *black* in different styles (100 % *solid*, 80 % *dashed*, 60 % *dotted*). Minimum and maximum denote respectively the lengths of the muscle when it is fully contracted and maximally stretched. **b** A typical force-velocity diagram (not to scale) for an isolated striated muscle [26]. −ve and +ve denote respectively negative and positive contraction velocities, the former corresponding to the shortening of a muscle and the latter to its elongating

Considering the pervasive importance that the phenomenon of the sticking point has either directly on competitive performance (e.g. in powerlifting) or on training performance and the ability to induce the desired adaptive stimulus, it is unsurprising that since the earliest observational work there have been attempts at explaining the reasons that cause this phenomenon to occur. Indeed it stands to reason that understanding the sticking point in terms of more primitive elements is important in informing the training and performance adjustments needed to overcome this bottleneck. These primitive elements include the anatomical cross-sectional area of a muscle [23], the force-length [24, 25] and force-velocity [26, 27] relationships (see Fig. 2a, b respectively), fatigue [28, 29], motor unit recruitment [30, 31], fibre type [32, 33], and biomechanical factors that affect torque development [34]. In seeking to explain the sticking point it is worth beginning with a point of universal consensus: for maximal lifts (i.e. lifts using the so-called one repetition maximum—the greatest resistance with which the trainee can complete a full repetition), muscular activation does not appear to be a significant contributor. Numerous studies using different exercises have consistently demonstrated that the prime movers are maximally activated from the very commencement of the lifting effort and remain so throughout the motion [4, 9].

### Biomechanical Disadvantage

Early attempts at explaining the occurrence of sticking points have largely concentrated on biomechanical factors. This includes biomechanical factors specific to a particular exercise as well as, inevitably, to a particular trainee such as limb length ratios [16]. In terms of the primitive elements that affect muscular force production, the focus here is on the structural mechanics that affect muscular torque transfer to the load and the force-length relationship characteristics of muscular force production.

For example, in their study of bench press performance by elite powerlifters, Elliott et al. [4] argue that the observed location of the sticking point is mostly explained by the mechanically disadvantageous position for the exertion of effective force against the load. Similar arguments were put forward by Madsen and McLaughlin [10] and Escamilla et al. [13] amongst others, and this explanation remains a popular one to this day [17, 36–38]. Nevertheless a more rigorous exploration of this claim readily exposes methodological flaws as well as inconsistencies. With regard to the former, it should be noted that Elliott et al. never actually investigated whether the position in question is indeed the biomechanically weakest one. As correctly noted by Kulig et al. [16] amongst others (also see Németh and Ohlsén [39], Arandjelović [19], and Bryanton et al. [40]), in multi-joint exercises such as the bench press, the effective strength curve is complex and involves a nonlinear combination of strength curves of individual muscles [19]. Indeed even in the simplest case of a single-joint exercise, because of the interplay between the force-length and force-velocity characteristics, and changing levers, the resulting strength curve is often not straightforward to predict as demonstrated by Blackburn and Morrissey in the example of leg extensions [21] and by Arandjelović in the example of arm curls [20] (also see work by Németh and Ohlsén [39]). Therefore the claim that a specific position in a lift is the weakest one requires empirical data, e.g. through a comparison with isometric strength characteristics. Although no data of this nature were provided by Elliott et al. [4], such a comparison in the context of deadlift performance was performed in detail by Beckham et al. [41] who found a poor match between points of isometric weakness and the observed sticking point when the exercise is performed in a conventional, dynamic fashion (though it should be noted that Beckham et al. performed allometric normalisation, which could have introduced artefacts in the data [42]).

Another observation that highlights the shortcomings of the purely biomechanical explanation concerns the changes in the sticking point locus across efforts of different intensities. As we already noted, in general the same individual will experience the sticking point at different stages in a lift taken to failure at different loads [3]. This could not be the case if biomechanics were the sole underlying factor.

### Decreased Passive Force

Motivated by the inadequacies of the purely biomechanical explanation of the sticking point, in recent years a number of researchers have sought to present an alternative theory that focusses on the changes in relative contributions of the passive and active components of muscular force. Recall that muscle force comprises an active component exerted by the contractive elements of the muscle and a passive component that is exerted by its non-contractile, structural elements [43, 44], both of which are dependent on the elongation of the muscle. Of particular importance here is the observation that the magnitude of the passive component of muscular force increases rapidly after a certain amount of stretch of the muscle has been reached. Considering that in most cases (though not universally) the main muscles involved in a certain lift experience the greatest stretch at the beginning of the exertion phase, the magnitude of passive muscular force decreases as the lift progresses. Hence, van den Tillaar and Ettema [5] argued that the sticking point emerges as a consequence of this decrease—if the force deficit is exhibited over a sufficient amount of time active contractions are insufficient to overcome the experienced external resistance and the lift fails at the sticking point. The finding that the maximal weight an athlete could lift is significantly reduced when the concentric action of prime movers is not preceded by an eccentric stretch [45] supports this hypothesis given that this effect of the so-called stretch-shorten cycle (SSC) [46–48] is thought to be effected by an elastic recoil of passive components of the muscle [49]. However this explanation too has failed to withstand empirical evidence, as acknowledged by van den Tillaar and Ettema themselves in their subsequent work [38, 50]. In particular there are several findings that speak against the decrease in passive force as the dominant factor in the development of the sticking point. For example, van den Tillaar et al. [38] observed that when it was not preceded by an eccentric portion, the sticking point on the bench press was higher than when the conventional execution of the exercise was adopted. This is contrary to what the proposed theory would predict—the presence of the stretch-shorten cycle would have been expected to result in delayed dissipation of the passive force contribution.

### An Overarching View

As we sought to illustrate, a number of challenges in the understanding of the phenomenon of the sticking point remain. This is not for lack of empirical data. The topic has received a remarkable amount of research attention and numerous well-designed studies in a variety of settings have been conducted; many of them are referenced in this article, and many others exist. Upon an examination of the corpus of relevant literature a disinterested researcher, we would argue, is led to the conclusion that much of the difficulty in trying to explain the sticking point is the result of the apparent desire to formulate an overly reductive yet universal model. It should come as no surprise that this may not be a realistic goal—different exercises in which sticking points are of interest are characterised by vastly different biomechanics (relative lever lengths, their changes over time, numbers of major contributing muscles, etc.), and different lifters exhibit different abilities (maximal force production, ability to sustain force, rate of force development, relative development of different muscle groups, etc.). These differences can greatly change the relative contributions of different elementary factors that contribute to the development of the sticking point: the dependence of the maximal voluntary force on muscle elongation and the speed of contraction, the elastic energy dissipated in the stretch-shortening cycle, the changes in internal and external levers, and fatigue. While in some circumstances one of these may indeed dominate, evidence suggests that this is not universally the case. The full understanding of the sticking point therefore requires a consideration of all of these factors and an explanation of a particular sticking point demands a thorough analysis of the particular lifter in the context in which the sticking point is observed.

Lastly it is worth nothing that research to date has very much focussed on what may be described as the impact of zeroth-order force dependence on the development of sticking points—both the force-length relationship and the torque effected by muscular force are factors that depend on the position in the lift only. In contrast the impact of the force-velocity (first-order force dependence) relationship on the sticking point has received little attention [20]. This is particularly surprising given that the development of momentum has been recognised as an especially important aspect in training and competition performance in practice [51], as we describe in further detail in the next section. The interplay of the aforementioned factors and fatigue, although noted by several authors [15, 52, 53], also demands more extensive study before its role in the context of sticking points is understood with some clarity and practical insight; notable research in this direction includes the work by Drinkwater et al. [2]. Finally, to the best of the authors’ knowledge the effect of muscular fibre type composition (both across individuals as well as across different exercises and muscle groups) on the occurrence and the location of sticking points has not been investigated at all.

## Training Strategies for Overcoming the Sticking Point

The phenomenon of a sticking point is multifactorial and underlain by complex interactions between different contributing factors that are both athlete-specific and exercise-specific. This makes the problem of addressing an athlete’s sticking point a major challenge in practice. A systematic approach is necessary—guided by empirical observations made in rigorous and controlled conditions reported in well-designed studies, a detailed analysis of an athlete’s performance should be used to identify the most promising training strategy. We identified five key strategies that a resistance training practitioner (coach or athlete) should understand and consider:

1. Target muscle strengthening using isolation work,
2. ROM-specific training using partial repetitions,
3. Development of momentum preceding the sticking point,
4. Exercise technique alteration, and
5. Accommodating or variable resistance use.

These are reviewed next—we explain the key ideas that motivate their use, outline how and when they should be applied, and highlight the target populations that they are most likely to benefit.

### Isolation Work

Many studies on the sticking point examined the stage in a lift at which the sticking point was observed for different exercises [6, 9, 13, 54, 55]. These findings can offer valuable insight into different strategies that can be employed to improve performance. In particular, by considering the biomechanical context (lever arms, elongation, etc.) in which different muscles contribute to the lift in the vicinity of the sticking point as well as the corpus of collected electromyography (EMG) data, in many cases it is possible to identify the muscle (or more broadly a functional muscle group) that can be considered the ‘weakest link’ [8, 20]. A straightforward application of this observation involves the strengthening of these muscles and especially so at the elongation at which failure occurs. Indeed power- and weightlifters have a long tradition of so-called assistance work, which accomplishes precisely this [8, 56, 57]. Common examples include the inclusion of chest isolation exercises by athletes who exhibit the sticking point at the onset of the concentric phase in the bench press [58] or the use of various isolation exercises for elbow extensors by athletes who encounter difficulties in the terminal stages of the lift [59].

### Partial Repetitions and Isometric Training

At the point in the ROM of a lift at which failure occurs, it can be readily seen that increasing the effective force that a trainee can exert against the load at this point will improve performance (note that this does not imply that the sticking point is the only or even the optimal such point, as discussed at length by Arandjelović [20]). Owing to the principle of specificity of strength adaptations [19, 60]—that is, the observation that the inducted adaptational stimulus to resistance exercise is the greatest for lifting conditions similar to those experienced during exercise—the most direct manner of addressing a sticking point is by employing partial repetitions [18] or isometric training [61, 62]. In particular, numerous studies have demonstrated that partial repetitions, whereby the load is lifted only through a limited part of the ROM in an exercise, is effective at increasing strength at approximately ±10°–20° from the trained joint angle [63, 64]. Similarly, functional isometrics that involve the application of force by the trainee against a load against a practically immovable obstacle (e.g. the pushing of a barbell against pins in a power rack) [65] have been shown to be successful at increasing strength at the specifically trained ROM [61, 62, 66, 67].

Considering the general consensus of empirical findings that suggest that partial and isometric training has limited potential for providing a sustained stimulus for muscular hypertrophy [62], these training modalities are of most direct interest to performance-oriented athletes. For athletes seeking increases in muscle mass the potential benefit may be indirect in that overcoming a specific sticking point may facilitate the use of greater loads in conventional training (which involves a combination of eccentric, concentric, and isometric contractions). However this potential value has to be carefully considered in the context of the invested time and effort, the associated neural fatigue, and psychological factors [68].

### Momentum

In Sect. 2 we noted that the sticking point in a lift may not necessarily occur at the point of greatest biomechanical disadvantage. For example, even if at a certain point in the ROM there is a net force deficit (i.e. the effective force an athlete is able to exert against the load is lower than the experienced external resistance), if the load has significant momentum the deficit may not effect a difficulty in overcoming this part of the motion. This observation leads to a popular training strategy employed by strength and power athletes that focusses on increasing force and its rate of development in the phases of a lift that precede a sticking point [69–71]. In particular so-called speed work involves the use of repeated low-intensity ($\approx 50$–60 % of one repetition maximum) sets, typically with short rest periods (45–60 s), with repetitions performed in a maximally accelerated fashion. This modality has been widely used by powerlifters [51, 69, 72, 73] in training for all three of the competition lifts (bench press, squat, and deadlift), and recent models described in the academic literature have started to elucidate the mechanisms underlying its effectiveness [20].

A different use of momentum for overcoming a sticking point involves the application of external momentum. In contrast to speed work training whereby the load is supplied momentum via the action of the muscles inherently involved in a particular exercise, external momentum is developed through the use of muscles otherwise not involved in a lift [14]. Though widely used by both recreational trainees and elite athletes [74, 75], this practice is often, if not usually, dismissed (as suggested by the morally loaded colloquial term ‘cheating’ used to describe it [76–79]) on the grounds that the use of excessive resistance increases the risk of injury and reduces the load experienced by the target muscles [76]. However recent models suggest that when used in moderation, external momentum can be safely used to apply greater force on target muscles as well as increase their time under tension (TUT) [14]. Considering safety and practical constraints (e.g. external momentum is easier to impart on isolation exercises, which generally involve the use of lighter loads), external momentum is of most use to athletes seeking increases in muscle size such as bodybuilders.

### Technique Alteration

The motion against resistance can be thought of as being effected by the sum of forces of muscles dynamically contributing to the lift, nonlinearly modulated by the given mechanical context [16]. Even when a single functional muscle group and its effects on motion around a single hinge joint are considered, the isolated characteristics of the effective force are greatly different from those of the muscle in isolation [20]. For complex multi-joint lifts, which involve a greater number of functional groups of muscles, the characteristics are far more multifaceted. This observation provides a powerful means of modifying a lift in a manner that eliminates or reduces the impact of a sticking point—by changing the style of exercise execution the biomechanical context can be changed. Distally speaking, this means that the points in the ROM at which a particular muscle are particularly strong (or weak) can be altered [4, 54], the time under tension (and with it fatigue) preceding the sticking point can be affected [8, 20] as well as the speed of contraction of contributing muscles at different points in the lift [19, 72]. In proximal terms the aim is to “flatten out” the difficulty of the lift [80]. Specific examples of how this may be achieved include alterations to the grip [54] or the stance [81, 82] width of the lifter, changes in the orientations of joint flexion/extension (or adduction/abduction) planes [3, 4, 82], adjustments in the synchronisation of movements across different joints [7], as well as numerous others [83].

It is important to stress that safety should always be an important consideration when attempting a modification of lifting technique. An unfamiliar biomechanical context itself can lead to injury so any changes should be done in a gradual fashion and using conservative loads until the lifter is familiarised with the newly adopted technique. In addition certain lifting styles may inherently carry certain risks, e.g. a wide grip on the bench press may increase the risk of shoulder injury and pectoralis major rupture [84], rounding of the back in the deadlift (which minimises the moment arm of the load around the hip) the risk of spinal injuries [85], and buckling of the knees (valgus collapse—poorly synchronised or excessive tibial internal rotation and adduction relative to the knee flexion angle in a given stance) in the squat the risk of knee injuries [86].

Exercise technique alterations are of most obvious utility to strength athletes whose primary aim is to complete a lift with the greatest amount of load, providing that the alterations are within the range permitted by their sport (e.g. see The International Powerlifting Federation [87] and The International Weightlifting Federation [88]). However employed in a targeted manner they can benefit a wide range of trainees. Bodybuilders for example may use them to place a greater emphasis on a certain muscle group (thereby possibly increasing the resistance experienced by the target muscles while reducing the total load lifted) while athletes may benefit from a style that is more suitable to their individual strengths and weaknesses and more effective at mimicking the manner in which they would perform a certain mechanical action.

### Accommodating and Variable Resistance

The term accommodating resistance refers to purposeful modifications of the effective load experienced in an exercise throughout a repetition [19, 89–92]. This technique is most often used in training by powerlifters [69] but also by other types of athletes in general strength and conditioning work [89, 91, 93]. One popular method of introducing accommodating resistance involves the fixing of an elastic band between the load (such as a barbell) and floor (or other fixed object, e.g. the power cage or the frame of a resistance machine). Typically, as the weight is lifted, the band is stretched and the resistance felt by the trainee increased [94–96]. Another commonly used alternative involves the use of heavy chains [97, 98], which are uncoiled and lifted off the floor during the lift thereby effecting an increase in resistance.

Both types of accommodating resistance are commonly recommended in powerlifting training for “overloading the top of the range of motion” [99–101] or increasing the rate of force development [93, 102, 103]. As such, when it comes to performance-oriented athletes (such as powerlifters), they are of most use in cases when the sticking point occurs in the terminal stages of a lift. For bodybuilders, or indeed other athletes looking to increase their muscle size, for whom the immediate aim is not the increase in performance in a particular exercise per se, the opposite prescription seems reasonable, i.e. an overload of the part of the ROM that is overcome easily. In this manner the entire ROM of an exercise can be made approximately uniformly challenging and closer to maximal resistance experienced throughout a set [80].

The mechanics of training aids such as elastic bands and chains limit the functional form of resistance alterations that can be achieved [104] (for a detailed review see Arandjelović [19]). Nevertheless other means of applying variable resistance are readily available in many training facilities [48, 104]. The most common ones include machines that achieve more complex loading patterns through the use of cams [80, 105], counterweights [106–108], and viscous resistance [104, 109]. The resistance modification achieved by each of these is quite different in nature: cams offer resistance variability as a function of the position in a lift, counterweights as a function of the acceleration of the load (i.e. the second derivative of position), and viscous resistance as a function of the speed of the load (i.e. the first derivative of position) [109]. By choosing an appropriate modification, which may include a combination of two or more of the aforementioned modalities, sophisticated effects can be achieved that best suit a particular athlete’s goals [109–111].

By varying the length of the moment arm of the force transmitted by the machine, cams allow a fixed force (the weight of the load) to produce a changing effective force experienced by the athlete. The force envelope is determined by the design, i.e. the shape of the cam [111]. One of the key ideas motivating the use of cams is that of attempting to match the resistive force of the machine with the force-length characteristics of human skeletal muscles [80, 104, 112, 113]. This would make them more suitable for hypertrophy-oriented athletes such as bodybuilders. The alteration of resistance characteristics through the use of counterweights is rather different in nature and may be described as reactive in the sense that the resistance is not dependent on the part of the exercise ROM per se but rather the instantaneous ease or difficulty of lifting exhibited by the trainee. As the detailed analysis presented by Arandjelović [109] demonstrated, at times when the load is moving with ease, i.e. with an increased acceleration of the load, the acceleration deficit between the load and the counterweights acts in a manner that increases resistance. The converse is true as well: when the acceleration of the load reduces, the effect of the counterweight is increased and the resistance felt by the trainee lessened, the least resistance being felt when acceleration reaches zero or becomes negative (as is the case in the exercise ROM preceding the sticking point) [109]. This is of most use to hypertrophy-oriented athletes for whom high tension sustained over time is crucial [114, 115].

## Summary and Conclusions

In this article we addressed a comprehensive range of issues that pertain to the so-called sticking points observed in resistance training. We made several important contributions of value to researchers and resistance training practitioners. We demonstrated that despite their nominal similarities and superficial resemblance, the spectrum of frequently adopted definitions of the sticking point describe significantly different phenomena, which has the potential to confound findings reported in the literature. Second we explained how only by considering the entire range of underlying physiological and biomechanical mechanisms can a particular sticking point be explained and understood. Using this insight we presented a range of different pertinent training strategies. We explained the key ideas that motivate their use, outlined how and when they should be applied, and indicated the target populations that they are most likely to benefit.

We trust that this work will serve to consolidate the existing body of work, direct future research, and instruct and inform strength practitioners using the most comprehensive body of evidence surveyed thus far.

## Footnotes

1 http://scholar.google.com.

2 http://www.ncbi.nlm.nih.gov/pubmed/.

## References

[1] van den Tillaar R, Ettema G (2009). A comparison of kinematics and muscle activity between successful and unsuccessful attempts in bench press. Med Sci Sports Exerc..

[2] Drinkwater EJ, Galna B, McKenna MJ (2007). Validation of an optical encoder during free weight resistance movements and analysis of bench press sticking point power during fatigue. J Strength Cond Res..

[3] Duffey MJ, Challis JH (2007). Fatigue effects on bar kinematics during the bench press. J Strength Cond Res..

[4] Elliott BC, Wilson GJ, Kerr GK (1989). A biomechanical analysis of the sticking region in the bench press. Med Sci Sports Exerc..

[5] van den Tillaar R, Ettema G (2010). The, “sticking period” in a maximum bench press. J Sports Sci..

[6] van den Tillaar R, Saeterbakken AT (2012). The sticking region in three chest-press exercises with increasing degrees of freedom. J Strength Cond Res..

[7] Hales ME, Johnson BF, Johnson JT (2009). Kinematic analysis of the powerlifting style squat and the conventional deadlift during competition: is there a cross-over effect between lifts?. J Strength Cond Res..

[8] McGuigan MR, Wilson BD (1996). Biomechanical analysis of the deadlift. Sports Med..

[9] Król H, Golas A, Sobota G (2010). Complex analysis of movement in evaluation of flat bench press performance. Acta Bioeng Biomech..

[10] Madsen N, McLaughlin T (1984). Kinematic factors influencing performance and injury risk in the bench press exercise. Med Sci Sports Exerc..

[11] van den Tillaar R, Andersen V, Saeterbakken AT (2014). The existence of a sticking region in free weight squats. J Hum Kinet..

[12] van den Tillaar R, Ettema G (2013). A comparison of muscle activity in concentric and counter movement maximum bench press. J Hum Kinet..

[13] Escamilla RF, Francisco AC, Fleisig GS (2000). A three-dimensional biomechanical analysis of sumo and conventional style deadlifts. Med Sci Sports Exerc..

[14] Arandjelović O (2013). Does cheating pay: the role of externally supplied momentum on muscular force in resistance exercise. Eur J Appl Physiol..

[15] García-López D, Herrero JA, Abadía O (2008). The role of resting duration in the kinematic pattern of two consecutive bench press sets to failure in elite sprint kayakers. Int J Sports Med..

[16] Kulig K, Andrews JG, Hay JG (1984). Human strength curves. Exerc Sport Sci Rev..

[17] Coburn J, Malek M. NSCA’s essentials of personal training. 2nd ed. Champaign: Human Kinetics; 2004.

[18] Massey CD, Vincent J, Maneval M (2004). An analysis of full range of motion vs. partial range of motion training in the development of strength in untrained men. Coll Antropol..

[19] Arandjelović O (2010). A mathematical model of neuromuscular adaptation to resistance training and its application in a computer simulation of accommodating loads. Eur J Appl Physiol..

[20] Arandjelović O (2011). Optimal effort investment for overcoming the weakest point - new insights from a computational model of neuromuscular adaptation. Eur J Appl Physiol..

[21] Blackburn JR, Morrissey MC (1998). The relationship between open and closed kinetic chain strength of the lower limb and jumping performance. J Orthop Sports Phys Ther..

[22] Cotterman ML, Darby LA, Skelly WA (2005). Comparison of muscle force production using the Smith machine and free weights for bench press and squat exercises. J Strength Cond Res..

[23] Fukunaga T, Miyatani M, Tachi M (2001). Muscle volume is a major determinant of joint torque in humans. Acta Physiol Scand..

[24] Zatsiorsky VM. Science and practice of strength training. Champaign: Human Kinetics; 1995.

[25] Komi P (1979). Neuromuscular performance: Factors influencing force and speed production. Scand J Sports Sci..

[26] Hill AV (1953). The mechanics of active muscle. Proc R Soc Lond B Biol Sci..

[27] Caiozzo VJ, Perrine JJ, Edgerton VR (1981). Training-induced alterations of the in vivo force-velocity relationship of human muscle. J Appl Physiol..

[28] Edwards RH, Hill DK, Jones DA (1977). Fatigue of long duration in human skeletal muscle after exercise. J Physiol..

[29] Jones DA, Bigland-Ritchie B, Edwards RHT (1979). Excitation frequency and muscle fatigue: mechanical responses during voluntary and stimulated contractions. Exp Neurol..

[30] Conwit RA, Stashuk D, Suzuki H (2000). Fatigue effects on motor unit activity during submaximal contractions. Arch Phys Med Rehabil..

[31] Hunter SK, Duchateau J, Enoka RM (2004). Muscle fatigue and the mechanisms of task failure. Exerc Sport Sci Rev..

[32] Bottinelli R, Pellegrino MA, Canepari M (1999). Specific contributions of various muscle fibre types to human muscle performance: an in vitro study. J Electromyogr Kinesiol..

[33] Johnson M, Polgar J, Weightman D (1973). Data on the distribution of fibre types in thirty-six human muscles: an autopsy study. J Exerc Physiol..

[34] Hoy MG, Zajac FE, Gordon ME (1990). A musculoskeletal model of the human lower extremity: the effect of muscle, tendon, and moment arm on the moment-angle relationship of musculotendon actuators at the hip, knee, and ankle. J Biomech..

[35] Wilkie DR. Studies in biology, No. 11: muscle. London: Edward Arnold; 1968.

[36] Colvin M. Quadriceps strength prediction equations in individuals with ligamentous injuries, meniscal injuries and/or osteoarthritis of the knee. [Master’s Thesis]. Auckland University of Technology; 2007.

[37] Dobbs CW. The effect of variable resistance training on lower limb strength and power development: a training study. [Master’s Thesis]. Waikato Institute of Technology; 2009.

[38] van den Tillaar R, Saeterbakken A, Ettema G. A comparison of muscle activition between maximum pure concentric and counter movement bench pressing. In: Proc annual conference of biomechanics in sports. 2012. p. 295–298.

[39] Németh G, Ohlsén H (1985). In vivo moment arm lengths for the hip extensor muscles at different angles of hip flexion. J Biomech..

[40] Bryanton MA, Kennedy MD, Carey JP (2012). Effect of squat depth and barbell load on relative muscular effort in squatting. J Strength Cond Res..

[41] Beckham GK, Lamont HS, Sato K (2012). Isometric strength of powerlifters in key positions of the conventional deadlift. J Trainology..

[42] Arandjelović O (2013). On self-propagating methodological flaws in performance normalization for strength and power sports. Sports Med..

[43] Hill AV (1938). The heat of shortening and dynamics constants of muscles. Proc R Soc Lond B Biol Sci..

[44] Verkhoshanksy YV, Siff MC. Supertraining. Denver: Supertraining Institute; 2004.

[45] Wilson GJ, Elliott BC, Wood GA. The effect on performance of imposing a delay during a stretch-shorten cycle movement. Med Sci Sports Exerc. 1991;23:364–370. 2020276

[46] Komi PV (2000). Stretch-shortening cycle: a powerful model to study normal and fatigued muscle. J Biomech..

[47] Doan BK, Newton RU, Marsit JL (2002). Effects of increased eccentric loading on bench press 1RM. J Strength Cond Res..

[48] Norrbrand L. Acute and early chronic responses to resistance exercise using flywheel or weights. Stockholm: Department of Physiology and Pharmacology, Karolinska Institutet; 2008.

[49] Hof AL, van den Berg JW (1986). How much energy can be stored in human muscle elasticity?. Hum Mov Sci..

[50] van den Tillaar R, Saeterbakken AT, Ettema G (2012). Is the occurrence of the sticking region the result of diminishing potentiation in bench press?. J Sports Sci..

[51] Young W (1993). Training for speed/strength: heavy vs. light loads. J Strength Cond Res..

[52] van den Tillaar R, Saeterbakken AT (2013). Fatigue effects upon sticking region and electromyography in a six-repetition maximum bench press. J Sports Sci..

[53] Arandjelović O. Computer simulation based parameter selection for resistance exercise. Model Simul. 2013:24–33.

[54] Wagner LL, Evans SA, Weir JP (1992). The effect of grip width on bench press performance. Int J Sport Biomech..

[55] McLaughlin TM, Dillman CJ, Lardner TJ (1977). A kinematic model of performance in the parallel squat by champion powerlifters. Med Sci Sports Exerc..

[56] O’Shea P (1985). The parallel squat. Strength Cond J..

[57] Burnett A, Beard A, Netto K. Back stress and assistance exercises in weightlifting. In: Proc Conference of international society of biomechanics in sports. 2002. p. 529–536.

[58] McLaughlin TN (1984). The biomechanics of powerlifting: assistance exercise, developing the chest and lats. Powerlift USA.

[59] Yarnell D. Forgotten secrets of the Culver City westside barbell club. SEATTLE: CreateSpace Independent Publishing Platform; 2011.

[60] Baker D, Wilson G, Carlyon B (1994). Generality versus specificity: a comparison of dynamic and isometric measures of strength and speed-strength. Eur J Appl Physiol..

[61] Graves JE, Pollock ML, Jones AE (1989). Specificity of limited range of motion variable resistance training. Med Sci Sports Exerc..

[62] Kitai TA, Sale DG (1989). Specificity of joint angle in isometric training. Eur J Appl Physiol Occup Physiol..

[63] Knapik JJ, Mawdsley RH, Ramos MU (1983). Angular specificity and test mode specificity of isometric and isokinetic strength training. Eur J Appl Physiol Occup Physiol..

[64] Thepaut-Mathieu C, Van Hoecke J, Maton B (1988). Myoelectrical and mechanical changes linked to length specificity during isometric training. J Appl Physiol..

[65] Jackson A, Jackson T, Hnatek J (1985). Strength development: using functional isometrics in an isotonic strength training program. Res Q Exerc Sport..

[66] O’Shea KL, O’Shea JP (1989). Functional isometric weight training: its effects on dynamic and static strength. J Strength Cond Res..

[67] Keogh JWL, Wilson GJ, Weatherby RE (1999). A cross-sectional comparison of different resistance training techniques in the bench press. J Strength Cond Res..

[68] Fleck SJ, Kraemer W. Designing resistance training programs. 4th ed. Champaign: Human Kinetics; 2014.

[69] Swinton PA, Lloyd R, Agouris I (2009). Contemporary training practices in elite British powerlifters: survey results from an international competition. J Strength Cond Res..

[70] Cronin J, McNair PJ, Marshall RN (2001). Developing explosive power: a comparison of technique and training. J Sci Med Sport..

[71] Gamble P (2006). Periodization of training for team sports athletes. J Strength Cond Res..

[72] Swinton PA. A biomechanical investigation of contemporary powerlifting training practices and their potential application to athletic development. [PhD Thesis]. Robert Gordon University; 2013.

[73] Jones MT (2014). Effect of compensatory acceleration training in combination with accommodating resistance on upper body strength in collegiate athletes. Open Access J Sports Med..

[74] Johnson M, Yesalis C (1986). Strength training and conditioning for wrestling: the Iowa approach. J Exerc Physiol..

[75] Gießing J, Preuss P, Fröhlich M. High-intensity post-exhaustion for maximizing training intensity in muscle hypertrophy training. In: Current results of strength training research: an empirical and theoretical approach. Göttingen: Cuvillier; 2005. p. 80–88.

[76] Algra B (1982). An in-depth analysis of the bench press. J Strength Cond Res..

[77] Stone WJ, Coulter SP (1994). Strength/endurance effects from three resistance training protocols with women. J Strength Cond Res..

[78] Miyaguchi K, Demura S (2006). Muscle power output properties using the stretch-shortening cycle of the upper limb and their relationships with a one-repetition maximum bench press. J Physiol Anthropol..

[79] Sundstrup E, Jakobsen MD, Andersen CH (2012). Muscle activation strategies during strength training with heavy loading vs. repetitions to failure. J Strength Cond Res..

[80] Folland J, Morris B (2008). Variable-cam resistance training machines: Do they match the angle-torque relationship in humans?. J Sports Sci..

[81] Escamilla RF, Fleisig GS, Lowry TM (2001). A three-dimensional biomechanical analysis of the squat during varying stance widths. Med Sci Sports Exerc..

[82] Escamilla RF, Francisco AC, Fleisig GS (2002). An electromyographic analysis of sumo and conventional style deadlifts. Med Sci Sports Exerc..

[83] Cholewicki J, McGill SM, Norman RW (1991). Lumbar spine loads during the lifting of extremely heavy weights. Med Sci Sports Exerc..

[84] Green CM, Comfort P (2007). The affect of grip width on bench press performance and risk of injury. J Strength Cond Res..

[85] Gardner PJ, Cole DE (1999). The stiff-legged deadlift. Int J Sports Med..

[86] Powers CM (2010). The influence of abnormal hip mechanics on knee injury: a biomechanical perspective. J Orthop Sports Phys Ther..

[87] The International Powerlifting Federation. The international powerlifting federation technical rules book. 2015. Available at: http://www.powerlifting-ipf.com. Accessed 4 Mar 2015.

[88] The International Weightlifting Federation. The international weightlifting federation technical and competition rules & regulation. 2015. Available at: http://www.iwf.net/. Accessed 4 Mar 2015.

[89] Berning JM, Coker CA, Briggs D (2008). The biomechanical and perceptual influence of chain resistance on the performance of the Olympic clean. J Strength Cond Res..

[90] Ebben WP, Jensen RL (2002). Electromyographic and kinetic analysis of backsquat variations. J Strength Cond Res..

[91] McCurdy K, Langford G, Ernest J (2009). Comparison of chain- and plate-loaded bench press training on strength, joint pain, and muscle soreness in division II baseball players. J Strength Cond Res..

[92] McMaster DT, Cronin J, McGuigan M (2009). Forms of variable resistance training. J Strength Cond Res..

[93] Anderson CE, Sforzo GA, Sigg JA (2008). The effects of combining elastic and free weight resistance on strength and power in athletes. J Strength Cond Res..

[94] Pizzari T (2008). Powerlifting and the art of elastic resistance training. Sport Health..

[95] Neelly K, Carter SA, Terry JG (2010). A study of the resistive forces provided by elastic supplemental band resistance during the back squat exercise: a case report. J Strength Cond Res..

[96] Kuntz CR, Masi M, Lorenz D (2014). Augmenting the bench press with elastic resistance: scientific and practical applications. J Strength Cond Res..

[97] Neelly K, Langevin SH, Jonathon BK (2010). The effects of an 8-week supplemental heavy chain resistance training program on lower extremity power in an elite athlete: a single-subject study. J Strength Cond Res..

[98] McCurdy K, Langford G, Jenkerson D (2008). The validity and reliability of the 1RM bench press using chain-loaded resistance. J Strength Cond Res..

[99] Neelly KR, Terry JG, Morris MJ (2010). A mechanical comparison of linear and double-looped hung supplemental heavy chain resistance to the back squat: a case study. J Strength Cond Res..

[100] Rhea MR, Kenn JG, Dermody BM (2009). Alterations in speed of squat movement and the use of accommodated resistance among college athletes training for power. J Strength Cond Res..

[101] Palmer TB. Electromyographic analysis of conventional and rubber-based band squats. [Master’s Thesis]. Texas State University-San Marcos; 2011.

[102] Baker DG, Newton RU (2009). Effect of kinetically altering a repetition via the use of chain resistance on velocity during the bench press. J Strength Cond Res..

[103] Neeld K. Rapid rate of force development. http://www.elitefts.com/education/training/rapid-rate-of-force-development/. Accessed Jan 2016

[104] Harman E (1994). Resistance training modes: a biomechanical perspective. J Strength Cond Res..

[105] Smith MJ, Melton P (1981). Isokinetic versus isotonic variable-resistance training. Am J Sports Med..

[106] Vingren JL. Mechanisms affecting bench press throw performance while using a counter-balanced Smith machine. [Doctoral dissertation]. University of North Texas; 2011.

[107] Buddhadev HH, Vingren JL, Duplanty AA (2012). Mechanisms underlying the reduced performance measures from using equipment with a counterbalance weight system. J Strength Cond Res..

[108] Kobayashi Y, Narazaki K, Akagi R (2013). Calculation of force and power during bench throws using a Smith machine: the importance of considering the effect of counterweights. Int J Sports Med..

[109] Arandjelović O (2012). Common variants of the resistance mechanism in the Smith machine: analysis of mechanical loading characteristics and application to strength-oriented and hypertrophy-oriented training. J Strength Cond Res..

[110] Vailas JC, Morris M, Pink M (1992). Muscle activity during isotonic, variable resistance, and isokinetic exercise. Clin J Sports Med..

[111] Amonette WE, Bentley JR, Lee SMC, et al. Ground reaction force and mechanical differences between the interim resistive exercise device (iRED) and Smith machine while performing a squat. NASA Johnson Space Center; 2004. NASA/TP-2004-212063, S-917, JSC-CN-8115.

[112] Wilson GJ, Elliott BC, Kerr GK. Bar path and force profile characteristics for maximal and submaximal loads in the bench press. Int J Sport Biomech. 1989;5:390–402.

[113] Johnson JH, Colodny S, Jackson D (1990). Human torque capability versus machine resistive torque for four Eagle resistance machines. J Exerc Physiol..

[114] Moritani T (1979). Neural factors versus hypertrophy in the time course of muscle strength gain. Am J Phys Med Rehabil..

[115] Tran QT, Docherty D, Behm D (2006). The effects of varying time under tension and volume load on acute neuromuscular responses. Eur J Appl Physiol..

